# Medical education beyond graduation: scientific initiation

**DOI:** 10.1590/S1516-31802010000300002

**Published:** 2010-05-06

**Affiliations:** I Full and associate professor, Department of Cardiopneumonology, Faculdade de Medicina da Universidade de São Paulo (FMUSP).; II Physician and postgraduate student, Discipline of Thoracic and Cardiovascular Surgery, Faculdade de Medicina da Universidade de São Paulo (FMUSP).

The increase in the number of medical schools active in Brazil has given rise to a discussion about undergraduate medical programs, with regard to what their precepts should be, what methodology would be most appropriate and which curricular model should be applied. Nonetheless, modern medical training can and must be more wide-ranging than the training normally envisaged in each school's curricular program. In this and in coming editorials, we will be discussing some of the so-called extracurricular activities that professors and institutions ought to be increasingly encouraging their students to participate in. We will begin with scientific initiation.

Scientific initiation can be defined as a tool for introducing undergraduate students to scientific research activities. It is the best opportunity for putting students into direct contact with scientific activity and engaging them in research.^1^ In practice, it consists of providing theoretical and methodological support for carrying out research projects.^2^

In this university-based activity, students have the opportunity to learn through taking on the role of researchers, under supervision. They can undertake all aspects of academic research: literature review, research design, practical development, academic writing and presentation of results in publications and scientific events.^3^

The specialized literature on medical education highlighted this topic starting in the 1990s, with emphasis on the importance of scientific issues in the general training for physicians.^4^ There is general agreement that the best way to teach students how to read and interpret scientific articles correctly, with critical analysis, is through experience of developing scientific projects.^5^ As done in other countries, several Brazilian medical schools have implemented programs of initiation to scientific research in the form of academic disciplines.^6,7^

Despite the importance of scientific initiation, it is still not offered to all undergraduate students in Brazil. In data from the Enade 2007 survey, 39% of the students stated that regulated scientific initiation existed in their courses; 12% said that scientific initiation existed, but in an unregulated form; 6.5% stated that scientific initiation existed, but was not integrated into the curriculum; 10.9% said that it was not offered; and 31% were unable to give an opinion.^8^

In addition to integration of scientific initiation into the undergraduate program, another major incentive indicated by students was the possibility of obtaining bursaries that are made available for project development. In Brazil, the National Council for Scientific and Technological Development (*Conselho Nacional de Desenvolvimento Científico e Tecnológico*, CNPq) is the main funding agency, through its Institutional Program of Scientific Initiation Bursaries (*Programa Institucional de Bolsas de Iniciação Científica*, Pibic). Nonetheless, there has been notable growth in the participation of state-based funding agencies, and the Research Support Foundation of the State of São Paulo (*Fundação de Amparo à Pesquisa do Estado de São Paulo*, Fapesp), the Research Support Foundation of the State of Rio de Janeiro (*Fundação de Amparo à Pesquisa do Estado do Rio de Janeiro*, Faperj) and the Research Support Foundation of the State of Minas Gerais (*Fundação de Amparo à Pesquisa do Estado de Minas Gerais*, Fapemig) have been prominent among these.

However, the presence of such bursaries should be thought of as fundamental for developing scientific initiation. Today, this has become a duty among institutions, rather than a sporadic activity. Because of the accelerated development of medical science and the constant avalanche of new information, it now has to be considered that scientific initiation should really become a basic element in physicians’ training. Scientific initiation bursaries should therefore be an individual incentive, as a form of selective funding for the best students, when they are linked to research projects.

The impact of scientific initiation programs on Brazilian national scientific production is not fully known. However, it can be indirectly estimated through the growing numbers of published papers that have studied this teaching activity and through the appearance of scientific meetings dedicated specifically to this activity, such as the National Scientific Initiation Congress, which has now been held eight times, and congresses developed by a variety of teaching institutions. Examples of the latter include the University Medical Congress of Universidade de São Paulo (USP), the Scientific Initiation Congress of Universidade de Brasília (UnB) and the Scientific Initiation Seminary of Pontifícia Universidade Católica, in Rio de Janeiro (PUC-Rio).

Another important point is the growing interest among the teaching staff in recruiting students for scientific initiation. The current model used by the Coordination Office for Advancement of University-level Personnel (Coordenação de Aperfeiçoamento de Pessoal de Nível Superior, Capes) has certainly had an influence, since the documents that describe the assessment criteria for postgraduate programs make two mentions of the importance given to scientific initiation with regard to guidance for students. Furthermore, lecturer incentive programs in several Brazilian universities have also ended up placing value on lecturers who are involved in scientific initiation. One example of this is the Strand A incentive program for academic production of FMUSP (Faculdade de Medicina da Universidade de São Paulo), which is managed by and under the responsibility of the Research Commission of FMUSP. Through this program, lecturers hired under a regime of exclusive dedication receive salary supplementation according to their academic and scientific production. These professors are assessed annually and guidance given to scientific initiation students scores points.

## CONCLUSION

Interest in scientific initiation in Brazil and around the world has been increasing, both among students and among professors and institutions. In Brazil, this has been propelled through incentives received through funding agencies, through the Capes model for postgraduate programs and, especially, through the growing need for up-to-date scientific knowledge on which to base day-to-day clinical practice.
